# Can biomarkers be used to diagnose attention deficit hyperactivity disorder?

**DOI:** 10.3389/fpsyt.2023.1026616

**Published:** 2023-03-08

**Authors:** Hui Chen, Yang Yang, Diana Odisho, Siqi Wu, Chenju Yi, Brian G. Oliver

**Affiliations:** ^1^Department of Pathology, The First Affiliated Hospital of Gannan Medical University, Ganzhou, China; ^2^School of Life Sciences, Faculty of Science, University of Technology Sydney, Ultimo, NSW, Australia; ^3^Research Centre, The Seventh Affiliated Hospital of Sun Yat-sen University, Shenzhen, China; ^4^Shenzhen Key Laboratory of Chinese Medicine Active Substance Screening and Translational Research, Shenzhen, China; ^5^Respiratory Cellular and Molecular Biology, Woolcock Institute of Medical Research, The University of Sydney, Glebe, NSW, Australia

**Keywords:** ADHD, behavioral, biomarkers, diagnosis, neuroscience

## Abstract

Currently, the diagnosis of attention deficit hyperactivity disorder (ADHD) is solely based on behavioral tests prescribed by the Diagnostic and Statistical Manual of Mental Disorders, 5th Edition (DSM-5). However, biomarkers can be more objective and accurate for diagnosis and evaluating treatment efficacy. Thus, this review aimed to identify potential biomarkers for ADHD. Search terms “ADHD,” and “biomarker” combined with one of “protein,” “blood/serum,” “gene,” and “neuro” were used to identify human and animal studies in PubMed, Ovid Medline, and Web of Science. Only papers in English were included. Potential biomarkers were categorized into radiographic, molecular, physiologic, or histologic markers. The radiographic analysis can identify specific activity changes in several brain regions in individuals with ADHD. Several molecular biomarkers in peripheral blood cells and some physiologic biomarkers were found in a small number of participants. There were no published histologic biomarkers for ADHD. Overall, most associations between ADHD and potential biomarkers were properly controlled. In conclusion, a series of biomarkers in the literature are promising as objective parameters to more accurately diagnose ADHD, especially in those with comorbidities that prevent the use of DSM-5. However, more research is needed to confirm the reliability of the biomarkers in larger cohort studies.

## 1. Introduction

Attention Deficit Hyperactivity Disorder (ADHD) is a behavioral disorder characterized by inattentiveness, hyperactivity, and impulsivity (1). It has a similarly high prevalence in both children and adults. In Australia, over 280,000 individuals aged between 0 to 19 years old, and over 530,000 adults aged 20 and older were affected by ADHD (2). This negatively affects working productivity with an estimated $20.4 billion in financial and wellbeing costs (2).

ADHD is currently diagnosed based on the individual’s behavior instead of any objective biomedical markers. The standard ADHD diagnosis is based on the Diagnostic and Statistical Manual of Mental Disorders, 5^th^ Edition (DSM-5), classified by the American Psychiatric Association (Table 1) (3). DSM-5 defines inattention, hyperactivity, and impulsivity as the main features of ADHD. Inattention refers to difficulty focussing on a job, wandering off task, absence of persistence, and disorganization (not due to lack of understanding). Hyperactivity refers to higher than normal levels of motor activity during an inappropriate setting, excessive fidgeting/tapping, and being talkative; while in adults, it can present as intense restlessness. Impulsivity refers to decision-making without forethought of long-term consequences and social intrusiveness, such as interrupting a conversation. Another example of impulsivity is accepting a job without carefully researching the position or considering the pros and cons of the job. Furthermore, DSM-5 diagnostic criteria for ADHD are used for patients with persisting symptoms for ≥6 months and in ≥2 settings, such as school, at home, or in a formal setting. The symptoms must not be better explained by other mental disorders/illnesses, e.g., anxiety disorder, bipolar disorder, personality disorder, or substance addiction/withdrawals, as shown in Table 1. Although this is the standard for ADHD diagnosis, many studies have ventured into researching biomarkers for ADHD, focusing on identifying the differences between an ADHD brain and a non-ADHD brain.

**Table 1:** Diagnostic criteria for attention deficit hyperactivity disorder as described in DSM-5 by the American Psychiatric Association.

Diagnostic criteria	
Ages 16 and below	Six or more symptoms persist ≥6 months, and directly negatively impact social activities and academics, from either one or both subtypes
Ages 17 and older	Five or more symptoms persist ≥6 months, and directly negatively impact social activities and academics from, either one or both subtypes
Subtypes of ADHD	
Inattention	Careless mistakes, lacks attention to detail
	Difficulty focussing/paying attention to tasks
	Difficulty listening when directly spoken to
	Lack of work completion due to difficulty following instructions
	Lacks organization skills, e.g., poor time management, messy
	Difficulty engaging in tasks which require consistent mental effort, e.g., schoolwork, preparing reports
	Often losing important personal property, e.g., wallet, mobile phones, school materials
	Easily distracted by irrelevant stimuli, whiles for adults it may involve extraneous thoughts
	Often forgetful throughout activities
Hyperactivity and Impulsivity	Often fidgeting, squirming, and tapping hands/feet
	Inability to stay seated
	Running and climbing during improper situations, but for adults it takes the form of restlessness
	Difficulty engaging in leisure activities in a quiet manner
	Acting as if “driven by a motor” or “on the go” e.g., uncomfortable to sit still for long periods of time
	Frequent excessive talking
	Difficulty waiting for their turn in social situations, interrupts conversation, intrudes on others, e.g., for adults it may be taking over other people’s tasks
	Difficulty waiting for their turn, e.g., queueing
	Frequently blurting out answers prior to question completion
Further criteria for diagnosis	
Symptoms were present prior to age 12	
Symptoms present in more than 2 settings	
Symptoms clearly lower quality of work whether in school, or at work	
Symptoms displayed cannot be better explained by another disorder or occur exclusively in another psychotic disorder	

Biomarker is a generic term for signs or characteristics that allows for accurate and objective diagnosis and monitoring of disease progression (4). They have been developed and are widely used for diagnosing and monitoring neurological disorders. There are four categories of diagnostic biomarkers that this review will use for ADHD – radiographic, molecular, physiological and histological markers. We based these upon diagnostic algorithms and/or the number of publications which we found. The most commonly used “biomarker method” in neuroscience is imaging after the discovery of distinct functional regions in the brain, also called “maps” (5). Several unique characteristics are found to be associated with ADHD, but require further research to become diagnostic biomarkers for ADHD. For example, the prefrontal cortex (PFC) was reported to be smaller in ADHD patients when scanned through magnetic resonance imaging (MRI) in several studies, with measurable features of neurodevelopmental disorders in those participants (6, 7). Moreover, previous reviews usually focus on one specific category of biomarkers, which suggested the potential of using specific electroencephalography (EEG) changes (8), or blood cortisol and inflammatory markers (9) as diagnostic biomarkers. However, no review has included the biomarkers of all four categories (radiographic, molecular, physiologic and histologic) that are commonly used in disease diagnosis. Therefore, this review assessed multiple biomarkers that researchers in the field may wish to combine in the future to increase the diagnostic/prognostic accuracy of ADHD, and to suggest the future direction of research on biomarkers to differentiate between an ADHD brain and a non-ADHD brain.

## 2. Potential biomarkers

A search on PubMed, Ovid Medline, and Web of Science was performed using “ADHD,” “biomarker,” “protein,” “blood/serum,” “gene,” and “neuro.” The search was performed by using “ADHD” and “biomarker” combined with one of “protein,” “blood/serum,” “gene,” and “neuro.” We included peer-reviewed original research papers published in English between Jan 2000 and Aug 2022, using either human subjects (diagnosed using DSM-5 or DSM-4) or animal models of ADHD (randomized trials, controlled trials, and observational studies) that reported positive results of investigated biomarkers. We excluded all the review articles, systematic reviews, meta-analyses, books or single-case studies which do not provide population-based information for the purpose of the development of future diagnostic methods. We included 92 original research studies, which are summarized in Table 2.

**Table 2:** Summary of findings.

Category	Methods	Biomarker/s	Sample Size	Age range (years)	Author, year
Radiographic	EEG	Less wave activity at low frequencies (approximately 1 Hz) during auditory Oddball and a visual continuous performance task in ADHD.	ADHD =175 Control = 175	6–18	Alexander et al., 2008 (10)
Radiographic Physiologic	EEG Actigraph	Significant correlation of attention with both hit reaction time interstimulus interval (ISI) change and hit standard error ISI change; Higher impulsive behavior and more target omissions in the continuous performance test in ADHD.	ADHD = 49 Control = 14	6.34–10.4	Chu et al., 2020 (11)
Radiographic	EEG	Greater frontal alpha during failed inhibition trials in ADHD.	ADHD = 25 Controls = 25	7–14	Ellis et al., 2017 (12)
Radiographic	EEG fNIRS	Decreased oxyhemoglobin in the right frontal cortex, as well as longer NoGo-P3 latencies and NoGo/Go-P3 amplitude.	ADHD = 20 Control = 19	8–11	Kaga et al., 2020(13)
Radiographic	EEG	Attenuated alpha power in ADHD; increased post- neurofeedback in resting-state alpha (i.e., rebound) in the ADHD, which correlates with individual improvements in motor inhibition (i.e., reduced commission errors).	ADHD = 25 Control = 22	Adults	Deiber et al., 2020(14)
Radiographic	EEG	Less stable brain arousal regulation with ADHD.	ADHD = 33 Control = 35	20–51	Strauss et al., 2018 (15)
Radiographic	EEG	Smaller amplitude of mismatch negativity in ADHD.	ADHD = 52 Control = 62	20.0–32.7	Hsieh et al., 2021 (16)
Radiographic	EEG and machine learning	rsEEG complexity of ADHD children was significantly lower than controls.	ADHD = 50 Control = 58	9.69–11.61	Chen et al., 2019 (17)
Radiographic	TMS-EEG	TMS evoked potentials (TEPs) and the ERP components in the Stop Signal task reduced in ADHD.	ADHD = 57 Control = 54	Mean 25.7 Mean 26	Hadas et al., 2021 (18)
Radiographic	EEG	The contralateral delay activity amplitude was attenuated in an early time window and the P3b was larger for ADHD.	ADHD = 16 Control = 16	17–48	Wiegand et al., 2016 (19)
Radiographic	EEG	Variability in stimulus event-related theta phase from frontal midline cortex strongly related to both ADHD, phenotypically and genetically.	67 twin pairs (34 monozygotic; 33 dizygotic)	8, 10, 12	McLoughlin et al., 2014 (20)
Radiographic	EEG	Mismatch negativity latency was shorter in ADHD; Mismatch negativity amplitude was higher in ADHD. Idiopathic 300 amplitude was lower in ADHD, with ADHD even lower than the Tuberous sclerosis complex ADHD patients.	Idiopathic ADHD = 13 Tuberous sclerosis complex ADHD = 6 Control = 14	7–17	Moavero et al., 2020(21)
Radiographic	EEG with machine learning	EEG/ERP-based neuroalgorithms are promising, but not sensitive enough to diagnose ADHD.	ADHD = 181 Control = 147	18–60	Müller et al., 2020 (22)
Radiographic	EEG	Attenuated ERP amplitudes and partly increased ERP latencies in ADHD.	ADHD = 447 Control = 227	6–60	Münger et al., 2021 (23)
Radiographic	EEG	The clustering coefficient is significantly higher, and the shortest path length is significantly lower in the beta band.	ADHD = 24 Control = 25	7–11	Dini et al., 2020 (24)
Radiographic	EEG	ADHD subjects manifested delayed developmental P3-trajectory in young adulthood and P3 reduction across all emotional valences.	ADHD = 45 Control = 41	18–59	Kakuszi et al., 2020 (25)
Radiographic	EEG	Lower theta/beta ratio in ADHD.	ADHD = 364	6–17	Snyder et al., 2015 (26)
Radiographic	EEG	Increased diffuse theta/beta power ratios with a widespread decrease in beta CSD in ADHD.	ADHD = 40 Healthy control = 41	8–16	Ahmadi et al., 2020 (27)
Radiographic	EEG	EEG-based indices using modeling	ADHD = 5 Control = 5	Adolescents unspecified	Modarres-Zadeh et al., 2005 (28)
Radiographic	EEG	Higher sleep spindle frequencies (12–14 Hz) in sleep stage 2 in ADHD.	ADHD = 21 Control = 18s	7.2–11.1	Saito et al., 2019(29)
Radiographic	EEG	Machine learning identifying EEG microstate. Decreased salience network (state C), increased duration and contribution of frontal–parietal network (state D), higher delta power in the fronto-central area and higher power of theta/beta ratio in the bilateral fronto-temporal area in ADHD brains. Unique EEG microstate across subtypes, predominant inattention (ADHD-I), and predominant hyperactivity-impulsivity (ADHD-HI), including ADHD-C having higher occurrence and coverage on the visual network (state B) than ADHD-I	ADHD-Is = 54 ADHD-Cs = 53 Control = 54	8–15	Luo et al., 2022 (30)
Radiographic	MRI	Cortical thickness	Participants from ADHD 200 database	Unspecified	Qureshi and Boreom, 2016 (31)
Radiographic	MRI	Widespread micro- and macrostructural changes of the brain in ADHD.	ADHD = 31 Control = 41	19.87–30.75	Gehricke et al., 2017 (32)
Radiographic	MRI	Increased macular thickness in the retina and increased ratio of thickness of the right frontal lobe to that of the parietal cortex.	ADHD = 12 Control = 13	Unspecified	Bae et al., 2019 (33)
Radiographic	MRI	Altered morphological connectivity included the insula, the caudal anterior cingulate cortex, the frontal pole, and the postcentral cortex in ADHD.	ADHD = 36 Control = 35	8.3–14.71	Wang et al., 2018 (34)
Radiographic	MRI	Ventromedial prefrontal cortex structure was negatively associated with multi-informant measures of ADHD symptoms.	2,223 adolescents	12.9–15.7	Albaugh et al., 2017 (6)
Radiographic	MRI	Reduced whole brain gray matter volumes in the right orbitofrontal cortex and bilateral hippocampus in boys with ADHD.	ADHD = 43 Control = 44	Unspecified	Liu et al., (35)
Radiographic	MRI	Reduced glutamate, N-Acetyl Aspartate and choline in the right PFC of ADHD brain.	ADHD = 21 Control = 15	8.36–11.54	Hai et al., 2020 (36)
Radiographic	MRI	Reduced brain iron levels in ADHD.	ADHD = 22 Control = 27	8–18	Adisetiyo et al., 2014(37)
Radiographic Physiological	MRI	Oversized left planum temporal and an abnormal interhemispheric asynchrony (10–40 ms) of the primary auditory evoked P1-response	ADHD = 73 Attention deficit disorder = 36 Dyslexics = 37 Control = 37	Unspecified	Serrallach et al., 2016 (38)
Radiographic	fMRI	Increased interhemispheric somatomotor functional connectivity and mirror overflow in ADHD.	ADHD = 62 Control = 57	8–12	Chen et al., 2021 (39)
Radiographic	fMRI	Functional connectivity of precuneus-post the salience network and dorsal default-mode network decreased in ADHD.	ADHD = 31 Control = 29	7.4–13.2	Wang et al., 2021 (40)
Radiographic	fMRI	Reduced inter-regional functional connectivity between right inferior fronto-frontal, fronto-striatal and fronto-parietal neural networks and parietal dysfunction in ADHD.	ADHD = 11 Control = 14	26–30	Cubillo et al., 2010 (41)
Radiographic	fMRI	Enhanced posterior putamen activation as a function of training in ADHD.	ADHD = 25 Neurotypicals =25	18–35	Ceceli et al., 2020 (42)
Radiographic	fMRI	Establishment of a brain mask to model brain function.	Participants from ADHD-200 database Training = 776 Test = 197	Unspecified	Dey et al., 2012 (43)
Radiographic	fMRI	Altered fractional anisotropies between the right inferior frontal gyrus and right superior temporal gyrus, right inferior frontal gyrus and right posterior cingulate, right anterior cingulate to posterior cingulate, and between left middle temporal gyrus (BA 39) and left posterior cingulate in ADHD.	ADHD = 60 Control = 16	10–18	Tremblay et al., 2020 (44)
Radiographic	fMRI a hybrid genetic algorithm	Using Metaheuristic Spatial Transformation to identify values in fMRI	Images from ADHD-200 database	Unspecified	Aradhya et al., 2020 (45)
Radiographic	NIRS	Reduced signals in the right and middle PFC in ADHD, compensated by the left PFC.	ADHD = 67 Control = 140	6.5–12.5	Yasumura et al., 2019 (46)
Radiographic	rfNIRS	Reduced brain signal variability in both high-order and primary brain functional networks with ADHD.	ADHD = 42 Control = 41	8–12	Hu et al., 2021 (47)
Radiographic	fNIRS	Both functional connectivity and global network efficiency decreased in ADHD.	ADHD = 30 Control = 30	7–12	Wang et al. 2020 (48)
Radiographic	fNIRS	Lower levels of arachidonic acid in children with ADHD.	ADHD = 24 Control = 22	8–14	Grazioli et al., 2019 (49)
Radiographic	fNIRS	Reduced activation in the right IFG/MFG during go/no-go tasks in ADHD.	ADHD = 16 Control = 16	6–14	Nagashima et al., 2014 (50)
Radiographic	fNIRS	Right prefrontal hypoactivation with ADHD.	ADHD = 30 Control = 30	6–15	Monden et al., 2015 (51)
Radiographic	fNIRS	Reduced activation in the right IFG/MFG during go/no-go tasks in ADHD.	ADHD = 16 Control = 16	6–13	Monden et al., 2015 (52)
Radiographic	NIRS	Lower oxygenated hemoglobin activity in the prefrontal region during the ‘to lose’ RPS task, particularly in the dorsolateral area	ADHD = 18 Control = 27	6–16	Ishii et al., 2017 (53)
Radiographic	SVM NIRS	RST interference with inhibitory control PFC oxy-Hb	ADHD = 108 Control = 145	Mean 10.6 Mean 9.69	Yasumura et al., 2020 (54)
Radiographic	TMS MRS	Reduced short interval cortical inhibition and in the dominant motor cortex and no correlation with γ-aminobutyric acid levels in ADHD.	ADHD = 37 typically developing (TD) peers = 45	8–12	Harris et al., 2021 (55)
Radiographic	pTMS	Atomoxetine treatment reduced pTMS-evoked SICI.	Participants = 14	8–16	Gilbert et al., 2007 (56)
Radiographic	Complex sound stimuli	Auditory Brain stem Response	ADHD = 33 Control = 40	26.5–49.2	Nielzén et al. 2016 (57)
Radiographic Molecular	fMRI RT-PCR	Decreased activation in parietal and (pre) frontal regions during response inhibition in ADHD.	ADHD = 20 Control = 38	Mean 14.1 Mean 13.26	Nielzén et al., 2016 (58)
Molecular	RT-PCR	Two quantitative trait loci (QTL), named Ofil1 (on chromosome 4) and Ofil2 (on chromosome 7).	Rats = 453	10 weeks	Vendruscolo et al., 2006 (59)
Molecular	rRT-PCR	Gene polymorphisms of DRD2, DAT1, IL-6, TNF-α, BDNF	ADHD = 119 Control = 153	7–13	Drtilkova et al., 2008 (60)
Molecular	RT-PCR	the 5’-UTR of dDAT1 gene.	ADHD = 14	6–12	Lambacher et al., 2020 (61)
Molecular	PCR ELISA	Auto-antibodies against dopamine transporter.	ADHD = 46 Control = 15	4–16	Lambacher et al., 2020 (62)
Molecular	rRT-PCR	AGO1 rs595961 was significantly associated with ADHD susceptibility	ADHD = 191 Control = 164	18–60	Karakas et al., 2017 (63)
Molecular	RT-PCR	*SNAP-25 MnlI* polymorphism (for evaluating the effects of Methylphenidate in children)	ADHD = 38	6.76–12.08	Li et al., 2022 (64)
Molecular	DNA methylation	Vipr2, Slc7a8, Mark2, Gart, Son	ADHD = 391 Control = 213	Mean 9.8	Mooney et al., 2020 (65)
Molecular	bioinformatics	Drd4 48 bp variants	ADHD = 3,000 Control = 16,000	Unspecified	Bonvicini et al., 2020 (66)
Molecular	DNA methylation	Drd4 methylation	Participants = 204	16–24	Cecil et al. 2018 (67)
Molecular	RT-PCR	Decreased expression of miR 18a-5p, 22-3p, 24-3p, 106b-5p and 107 and increased expression of miR 155a-5p in ADHD; miR-107 may be a candidate biomarker	ADHD = 50 Control = 50	7–17	Kandemir et al., 2014(68)
Molecular	NGS RT-PCR	MiR-140-3p, miR-let-7 g-5p, miR-30e-5p, miR-223-3p, miR-142-5p, miR-488-5p, miR-151a-3p, miR-151a-5p, miR-126-5p in total white blood cells were significantly upregulated in ADHD.	ADHD = 88 Control = 74	6.9–12.7	Wang et al., 2018 (69)
Molecular	RT-PCR	MiR-140-3p, miR-27a-3p, miR-101-3p, let-7 g-5p, miR-30e-5p, miR-151-3p, miR-142-5p, miR-150-5p, miR-151a-5p, miR-223-3p, miR-486-5p in total white blood cells were significantly upregulated in ADHD.	ADHD = 145 Control = 83	6–12	Wang et al., 2022 (70)
Molecular	RT-PCR	Higher serum miR-let-7d level in ADHD	ADHD = 35 Control = 35	6–14	Wu et al., 2015 (71)
Molecular	RT-PCR	Higher serum miR-let-7d level in ADHD	ADHD (non-drug group) = 43 ADHD (drug group) = 32 Control = 18	6.3–11.43	Cao et al., 2019 (72)
Molecular	Epigenome-wide association study	Methylation quantitative trait loci within *SLC7A8* and *MARK2*.	ADHD = 391 Control = 213	7–12	Mooney et al., 2020 (65)
Molecular	DNA methylation	Reduced global DNA methylation (5-mc) in ADHD.	ADHD = 394 Control = 390	Mean 34.02 Mean 29.36	Müller et al. 2022 (73)
Molecular	DNA methylation	*Lime1*, *Sptbn2*	ADHD = 126 Control = 72	6–16	Sung-Chou Li et al. 2021 (74)
Molecular	DNA methylation	*MAD1L1*, *MGC87042*, *PTPRN2*, and *SGK2*	ADHD = 22 Control = 54	4–19	Goodman et al., 2020 (75)
Molecular	DNA methylation	Methylation levels of *SLC6A3* gene, coding for the human dopamine transporter (DAT) greatly reduced in ADHD.	ADHD = 30 Control = 15	6–14	Adriani et al., 2018 (76)
Molecular	PCR	*KIAA0319, FOXP2*, and *DCDC2*-*KIAA0319* SNPs correlated with dyslexia; *DCDC2*-*DYX1C1* SNPs associated with ADHD; *COMT1, MAOA, DBH* SNPs correlated with different dysfunctions.	ADHD/Dyslexia = 2078 Control = 3,357	Unspecified	Sanchez-Moran et al., 2018 (77)
Molecular Radiographic	DNA methylation PET	Norepinephrine transporter promoter was hypermethylated in ADHD. A negative correlation between methylation of a CpG site and norepinephrine transporter distribution in the thalamus, locus coeruleus, and the raphe nuclei.	ADHD = 23 Control = 23	20.3–43.1	Sigurdardottir et al., 2021 (78)
Molecular	RT-PCR	Telomere length.	61 ADHD children and their parents	6–16	Costa Dde et al., 2015 (79)
Physiological	Pupillometric	Smaller pupil size in ADHD.	ADHD = 28 Control = 22	Unspecified	Das and Khanna, 2021 (80)
Physiological	Eye tracker	Larger pupil diameters and lower temporal complexity and symmetry in ADHD	ADHD = 16 Control = 20	22–51	Sou Nobukawa et al., 2021 (81)
Physiological	Pupillometric	A decreased pupil diameter during a spatial working memory task. Pupil size correlated with the subjects’ performance and reaction time variability.	ADHD = 28 Control =22	10.17–12.05	Wainstein et al., 2017 (82)
Physiological	Observation	Small difference in eye convergence response	ADHD = 108 Control = 36	18–65	Jimenez et al., 2021 (83)
Physiological	Salimetric	Lower levels of awaking salivary cortisol and BDNF in ADHD.	ADHD = 98 Control = 21	6–18	Chang et al., 2020 (84)
Physiological	Observation	Perinatal Pitocin exposure is a risk factor for ADHD.	Childbirth records = 172	3–25	Kurth and Haussmann, 2011 (85)
Physiological	Atomic absorption spectroscopy X-ray fluorescence	Higher blood-lead levels correlate with higher hyperactivity/impulsivity scores; Higher bone-lead levels correlate with both higher hyperactivity/impulsivity and oppositional-defiant-disorder scores.	ADHD = 164	3–15	Lin et al., 2019 (86)
Physiological	LC–MS	Biomarkers of oxidative stress, such as 4-hydroxy-2-nonenal-mercapturic acid (HNE-MA) showed higher in ADHD.	ADHD = 76 Control = 98	4–15	Waits et al., 2022 (87)
Physiological	LC–MS/MS	Lower blood anthranilic acid and higher blood tryptophan and kynurenine levels in ADHD.	ADHD = 102 Control = 62	6–16	Evangelisti et al., 2017 (88)
Physiological	chemiluminescent immunometric assay	Lower serum Triiodothyronine levels with ADHD.	ADHD = 30 Control = 30	6–15	Kuppili et al., 2017 (89)
Physiological	ELISA	Decreased serum adiponectin levels in ADHD.	ADHD = 44 Control = 29	Median (first, third quartile) 25 (23, 29.8) 24 (22, 27.5)	Mavroconstanti et al., 2014 (90)
Physiological	K-SADS-E	Decreased serum levels of dehydroepiandrosterone sulphate (DHEA-S) in ADHD.	ADHD (Boys) = 113 ADHD (Girls) = 35 Control (Boys) = 46 Control (Girls) = 26	6.3 ± 11.7	Wang et al., 2019 (91)
Physiological	LC–MS/MS	A reduction in serum levels of anthranilic acid and tryptophan in ADHD.	ADHD = 102 Control = 62	6.6–12.0	Evangelisti et al., 2017 (88)
Physiological	LC–MS/MS	Lower levels of all major serum sphingomyelins (C16:0, C18:0, C18:1, C24:1, ceramide C24:0, and deoxy-ceramide C24:1) with ADHD.	ADHD = 28 Related controls = 28 Non-related controls = 21	5–18	Henríquez-et al., 2015 (92)
Physiological	PUFA biostatus fNIRS	Lower blood levels of arachidonic acid in ADHD.	ADHD = 24 Control = 22	8–14	Grazioli et al., 2019 (49)
Physiological	Receptor Binding Assay	Decreased binding capacity of muscarinic acetylcholine receptors in fibroblasts in boys with ADHD.	ADHD = 11 Control (unspecified)	7–12	Johansson et al., 2013 (93)
Physiological	LC–MS	Maternal plasma biomarkers of acetaminophen.	ADHD = 188	Unspecified	Ji et al., 2018 (94)
Physiological	MS	High maternal blood selenium levels predict risk of ADHD.	ADHD = 216 Control = 651	Unspecified range	Lee et al., 2021 (95)
Physiological	GC–MS Behavioral studies	Ethyl glucuronide value showed a positive correlation between ADHD-related behavior.	Unspecified	6–9	Eichler et al., 2018 (96)
Physiological	Microbiome	*Sutterella stercoricanis* and *Bacteroides uniformis* positively correlates to ADHD symptoms.	ADHD = 30 Control = 30	Mean 8.4 Mean 9.3	Wang et al., 2020 (97)

ADHD: attention deficit hyperactivity disorder; BDNF: brain derived neurotrophic factor; “COS,” complementary on the opposite strand; DAT1: dopamine transporter; EEG: electroencephalography; ELISA: enzyme linked immunosorbent assay; ERP: event related potential; fMRI, functional magnetic resonance imaging; fNIRS: functional near-infrared spectroscopy; HNE-MA: 4-hydroxy-2-nonenal-mercapturic acid; IFG/MFG: inferior and middle prefrontal gyri; LC–MS: Liquid Chromatography-Mass Spectrometry; LC–MS/MS: liquid chromatography–tandem mass spectrometry; MS: Mass spectrometry; MRS: magnetic resonance spectroscopy; MRI: magnetic resonance imaging; N/A: Not Applicable; NGS: next-generation sequencing; NIRS: near-infrared spectroscopy; oxy-Hb, oxygenated hemoglobin; PFC: prefrontal cortex; pTMS: paired-pulse transcranial magnetic stimulation; rsEEG: resting-state EEG; RST: reverse Stroop task; SICI: short interval cortical inhibition; SVM, support vector machine; RT-PCR: reverse transcription-polymerase chain reaction; TMS: transcranial magnetic stimulation; UTR: untranslated region; PET: positron emission computed tomography.

The majority of the studies were on human participants and included age-matched controls for the ADHD participants to allow for non-biased comparison. Studies using existing databases (31, 43, 45) did not report age range. Some studies did not specify age ranges, but attempted to match the sample size of ADHD and control participants (28, 35, 38, 77, 80, 95). Several studies did not compare ADHD participants with controls. One assessed the participants with either high or low levels of ADHD traits using an adult ADHD self-report scale following the DSM-IV criteria (98). Some studies performed differential diagnosis with an integration method criterion E to help improve diagnostic accuracy (9, 26), or inform stratification of patients to their individual best treatment and enhance remission rates (64, 99). A study revealed the correlation between ventromedial prefrontal volume and multi-informant measures of ADHD symptomatology in a large population-based sample of adolescents (6). The age range of the participants varies between studies; however, the majority of the participants were under the age of 18, as ADHD is more common in the younger population, and the symptoms tend to improve with development (100). As shown in Table 2, most studies focused on participants aged between 6 to 18 years old. A total of 15 studies specified adult participants aged from 16 to 65. Eleven studies did not specify the age ranges of their participants in their recruitment methods. One study used a 10-week-old rat model.

Selected articles were categorized into four types of biomarkers: radiographic, molecular, physiological, and histological markers.

### 2.1. Radiographic markers

There were 51 papers reported using the radiographic techniques alone or with other approaches to identify the biomarkers of ADHD, including EEG, MRI, and near-infrared spectroscopy (NIRS) (Table 2). These methods were all noninvasive, which is preferable for ADHD diagnosis, given most individuals with ADHD are children at the time of diagnosis.

#### 2.1.1. EEG

EEG is typically used to diagnose sleep disorders and seizure activities during epilepsy by attaching electrodes to the scalp to record electrical activity in the brain. We found 20 studies in the database using EEG methods to identify radiographic biomarkers that can be used to diagnose ADHD (Details in Table 2). During the Oddball task (an auditory experiment) and Visual Continuous Performance Task, ADHD participants showed fewer wave activities at low frequencies (~1 Hz) than the controls, which correlates to hyperactivity or increased levels of impulsivity (10). There were higher impulsive behavior and more target omissions in the continuous performance test in patients with ADHD (11). During failed inhibition trials, ADHD youth displayed greater frontal alpha asymmetry than controls youth (12). Decreased oxygenated hemoglobin concentrations (Oxy-Hb) in the right frontal cortex were found in children with ADHD using functional near-infrared spectroscopy (fNIRS) and EEG (13). Moreover, cortical hyperactivation results from reduced resting alpha power using EEG neurofeedback study in adults with ADHD (14). Adult ADHD patients showed significantly lower arousal levels and substantially less stable brain arousal regulation than controls (15). Compared with adult controls, it was also observed that a smaller amplitude of mismatch negativity and its differential associated pattern with inattention, real-world executive dysfunction, and poor decision-making ability in drug-naive adults with ADHD (16).

Furthermore, it has been suggested that the combination of EEG characteristics obtained by various methods can perform better in identifying ADHD than using a single type of feature (17). Hadas et al. (18) used transcranial magnetic stimulation (TMS) evoked potentials (TEPs) and event-related potentials (ERPs) in EEG and discovered an association between reduced right lateral PFC excitability and the severity of ADHD symptoms. In addition, ERP assessed during cognitive tests was found to correlate to a reduction in visual short-term memory, which can be used as another diagnostic biomarker for ADHD (19). Specifically, one type of ERP, P3b, was much higher in those diagnosed with ADHD when compared to the control participants (19). Another study assessed 67 twin pairs (34 monozygotic, 33 dizygotic) with concordance or discordance for ADHD symptomatology with age-matched controls, where variability in stimulus event-related theta phase signals at the frontal midline cortex strongly correlates to ADHD, both phenotypically and genetically (20). A study by Luo et al. (30) using machine learning to identify EEG microstate also found higher delta power in the fronto-central area and higher power of theta/beta ratio in the bilateral fronto-temporal area in ADHD brains (p. 106). Among patients with ADHD, those with tuberous sclerosis complex (TSC) often have more severe symptoms than idiopathic cases (21). ERP can distinguish these two types, where idiopathic 300 amplitude is lower in ADHD patients compared with the controls, with idiopathic ADHD even lower than Tuberous sclerosis complex ADHD patients (21). In the context of a standard Go/NoGo task (visual continuous performance test [VCPT]), attenuated ERP amplitudes, and increased ERP latencies in ADHD subjects compared to controls were also reported (23). However, a study also shows that ERP-based algorithms generated by machine learning are not sensitive enough to diagnose ADHD (22), suggesting that a better coding system may be needed. In the study by Luo et al. (30) used different machine learning algorithms which identified unique resting state EEG microstates across two subtypes of ADHD in young patients, predominant inattention (ADHD-I) and predominant hyperactivity-impulsivity (ADHD-HI). While all ADHD brains showed decreased salience network (state C) and increased duration and contribution of frontal–parietal network (state D), patients with ADHD-C had higher occurrence and coverage on the visual network (state B) than those with ADHD-I (30). This method may be useful in accurately distinguishing the ADHD sub-types.

A study combined the recognition of facial emotion task and EEG to assess children with ADHD and age-matched healthy controls, where beta band abnormalities correlate with disorders in processing facial emotions (24). Across all emotional valences, ADHD subjects manifested delayed developmental P3-trajectory in young adulthood and P3 reduction (25). Interestingly, the study by Snyder et al. (26) demonstrated that the theta/beta ratio in EEG assessment is a reliable potential biomarker for ADHD diagnosis by improving the diagnostic accuracy from 61% to 88%. They found that approximately a third of their participants showing normal EEG were wrongly diagnosed with ADHD after being reassessed by clinicians using the DSM-5 criteria because the clinicians specifically focussed on criteria E in Table 1, i.e., symptoms cannot be better explained by another disorder (26). In fact, 91% of wrongly diagnosed participants showed lower theta/beta ratios, whereas those with ADHD showed higher theta/beta ratios (26, 27). This also further reinforces the need for more accurate diagnostic criteria to minimize the chance of misdiagnosis, so the children can receive the correct care during their critical mental development period.

In the study by Modarres-Zadeh et al. (28), the auditory-based attention tests and EEG were combined to give results in the form of indices; those with ADHD were tested immediately before and then an hour after taking ADHD medication. The index of ADHD participants was different from that of non-ADHD participants, which was improved after the medication (28), suggesting its potential as a treatment surveillance tool. Children with ADHD are reported to be more prone to have difficulties in sleep during the night (101). It was reported that slow-spindle activity was enhanced in ADHD subjects (29). However, both studies need to be confirmed by larger size studies (28, 29).

#### 2.1.2. MRI

Regular MRI can provide information on structural changes in specific brain regions. Nine studies using regular MRI scanning have suggested distinct brain volume changes in patients with ADHD (Table 2). Using the largest open-source MRI database ADHD-200 dataset, cortical thickness in the temporal junction and insula were found to be reduced by 7–8 and 7%, respectively, in the ADHD-affected brains compared to the controls (31). More structural brain anatomy and connectivity, such as maturation of white matter fiber bundles, gray matter density (32), the right frontal lobe (33), the insula, the caudal anterior cingulate and postcentral cortex (34), associated with an ADHD diagnosis were reported in other studies. In a large population-based study on adolescents (n = 1,538), a negative correlation was found between ventromedial PFC volume and self-reported ADHD symptom severity and reaction time variability (6). The volumes of the right orbitofrontal cortex and bilateral hippocampus are also reduced in young males with ADHD (35). Another study suggested the potential of combining several MRI T1-weighted features to better classify ADHD patients, including surface values, gray matter volumes, and radiomic features (102). In another study, reduced neurochemical signals, such as glutamate, N-Acetyl Aspartate and choline, were reported in the right PFC of ADHD (36).

Functional MRI (fMRI) can identify abnormal activities and connectivity between brain regions in ADHD brains, which were used in eight papers (Table 2). Increased interhemispheric somatomotor functional connectivity and mirror overflow in ADHD (39), while functional connectivity of precuneus-post the salience network and dorsal default-mode network (DMN) decreased in the ADHD (40). During Stop Signal Task and Cognitive Switch Tasks, lower activation was observed in several brain regions of adults with childhood ADHD, including the PFC, caudate and thalamus during both tasks, and the left parietal lobe during the Switch task, with reduced functional connectivity between right inferior fronto-frontal, fronto-striatal and fronto-parietal neural networks (41). A negative correlation was found between symptom severity and hyper-activation in fronto-striatal, parietal and cerebellar brain areas (41, 103). While training on two stimulus–response-outcome associations, the ADHD group displayed a distinct neural signature marked by enhanced posterior putamen activation (42). Tremblay et al. (44) used fMRI to study reduced inhibitory control in white matter during Stop Signal Task in ADHD participants and found abnormal activities in the tracts connecting the network nodes of part of the inferior frontal-occipital fasciculus and cingulum. Participants with ADHD showed altered fractional anisotropies between the right inferior frontal gyrus and right superior temporal gyrus (*p* = 0.09 vs. control), right inferior frontal gyrus and right posterior cingulate (*p* = 0.01 vs. control), right anterior cingulate to posterior cingulate (*p* = 0.08 vs. control), and between left middle temporal gyrus (BA 39) and left posterior cingulate (*p* = 0.02 vs. control) (44) Although the small sample size (ADHD *n* = 60, control *n* = 16) limits the power to detect statistical significance in some comparisons, the results suggest the potential of using these tracts in the white matter to diagnose deficiency in inhibitory control among patients with ADHD. In addition, there are disintegrated activations of the auditory complex in people with ADHD and dyslexia that can be normalized by regularly playing a musical instrument; however, currently, this parameter is not distinguishable between ADHD and dyslexia, therefore not suitable for diagnosis but initial screening (38). Another study showed dysconnectivity between the default-mode network and the salience network in ADHD brains, which may serve as a useful diagnostic biomarker (40). While individual clinical studies correlate behavioral tests and fMRI changes using a relatively small sample size, Dey et al. (43) applied machine learning to a large number of resting fMRI images (*n* = 776) from the ADHD-200 database to create a more comprehensive network called “brain mask”. They identified ADHD-specific changes in the neuronal connectivity in the cingulate gyrus and paracingulate gyrus in images of ADHD brains, which may be potentially developed into a diagnostic tool with high accuracy (43). Using the same database, another group used a different algorithm called “metaheuristic spatial transformation” to analyse the resting fMRI images, which significantly increased the accuracy of ADHD diagnosis, especially distinguishing it from autism (45). It can be predicted that artificial intelligence will be applied to such imaging based diagnoses in the near future, not limited to ADHD.

Multimodal MRI using machine learning [e.g., heterogeneous graph attention convolutional network by Yao et al. (104)] can integrate the brain activities imaged by both fMRI and diffusion MRI (dMRI) to increase the efficiency of capturing ADHD-related brain features. This attempt may lead to future automated diagnoses by artificial intelligence. Moreover, multimodal MRI can provide information on abnormal metabolism in the brain tissue. A study using this tool showed that the iron levels in the striatal and thalamic areas were considerably decreased in medication-naïve ADHD participants; whereas, in ADHD participants receiving medications, the iron levels were similar to the controls (37). This suggests that brain iron level changes may be a plausible biomarker to monitor the response to ADHD medication; however, such a technique can be costly for most patients.

#### 2.1.3. NIRS

NIRS is used to measure regional oxygenation in brain regions, which was used in 10 papers (Table 2). ADHD participants showed reduced signals in the left, right and middle PFC, suggesting abnormal hemodynamics (46–48). Using a support vector machine, the sensitivity and specificity of NIRS to differentiate ADHD and controls can reach 83.78% and 88.71%, respectively (54). A study has correlated reduced blood levels of polyunsaturated fatty acids (another possible biomarker of ADHD) and low signal in functional NIRS (fNIRS) during complex tasks in children with ADHD, suggesting the feasibility of integrating such measurements with behavioral tests to more accurately diagnose ADHD (49). This study also suggested that abnormal neurodevelopment in children with ADHD may be due to reduced oxygenated and deoxygenated hemoglobins and hemodynamic shifts in the frontoparietal region (49). In addition, the inferior and middle frontal gyri were found to generate the most discriminative signals between ADHD and control brains (51). During go/no-go or “rock, paper, scissors” (RPS) tasks, oxyhemoglobin signals assessed by fNIRS were also reduced in the right inferior frontal gyrus/middle frontal gyrus and border of inferior and middle frontal gyri in medication-naïve ADHD participants (13, 52, 53). However, such abnormalities in fNIRS signals can be normalized by the AHDH treatment, selective norepinephrine reuptake inhibitor atomoxetine (50), suggesting the potential of using fNIRS for treatment surveillance. To further establish valid and objective biomarkers for ADHD with data collected with NIRS, the machine learning method was used to predict children with ADHD using prefrontal cortex activity in a multicenter study (54). The sensitivity and specificity were all relatively high, reaching 88.71% and 83.78%, respectively, suggesting its potential to be used as a diagnostic tool for children with ADHD.

#### 2.1.4. Other methods

A study using transcranial magnetic stimulation (TMS) showed reduced short interval cortical inhibition in the dominant motor cortex (55). It also used magnetic resonance spectroscopy (MRS) to measure the response of γ-aminobutyric acid (GABA), the inhibitory neurotransmitter (55). Although they found a negative correlation between short interval cortical inhibition and GABA response, GABA level is not linked to ADHD symptoms. One study linked fMRI imaging to the genetic endophenotypes of ADHD participants, who displayed lower activation in the parietal and prefrontal regions (58). One study used paired-pulse TMS to determine the treatment efficacy of atomoxetine in participants with Tourette syndrome and ADHD, which showed that improved ADHD scores were due to decreased short interval cortical inhibition by atomoxetine (56). In a study on adults 26.5–49.2 years of age, highly significant differences from healthy subjects occurred in the Auditory Brainstem Response of ADHD cases (57). The findings of these studies are interesting by providing potential mechanisms for ADHD pathogenesis; however, they may have limited value in diagnosis.

### 2.2. Molecular biomarkers

The molecular studies often require blood samples, and/or buccal swabs, from the participants to assess their gene changes, which is considered a reasonable method. There were 21 papers that examined molecular markers alone and two studies in combination with imagining analysis (Table 2). Polymerase chain reaction (PCR) is the most common method for gene-related assays, and seven studies reported the outcome using PCR or real-time quantitative reverse transcription PCR (qRT-PCR) (Details in Table 2.). DAT auto-antibody levels were higher in individuals with ADHD, which can be normalized after methylphenidate treatment (62). In another study, atypical genotypes (8/10, 7/10 and 10/11) of the dopamine transporter gene (DAT1) gene were also found in hyperkinetic boys (105). However, a later study showed that the polymorphism of the DAT1 gene does not correlate with ADHD (106). Recently, more polymorphism in dopamine transporter genes have been reported in patients with ADHD, such as AGO1 rs595961 (63), *SNAP-25 MnlI* (64) and dopamine receptor D4 (DRD4) (9, 66), suggesting the role of gene variants in the pathogenesis of ADHD. In addition, changes in allelic and genotype frequencies of the TaqI A polymorphism of the dopamine receptor D2 (DRD2) gene were found in ADHD patients, with the frequency of the allele A1 higher in the hyperkinetic boys compared to the controls (77). The polymorphisms of genes involved in neuroinflammation (IL-6 and TNFα) and neurodevelopment (brain-derived neurotrophic factor) were also found in boys with hyperkinesia, which were associated with their neurological performance (77). However, changes in the expression of IL-6, TNFα and BDNF are also found in other neurological conditions, which may not be specific enough for ADHD.

With the development of technology, epigenetic modification (such as DNA methylation and microRNA analyses) has gained attention in the pathogenesis of ADHD. MicroRNAs are small non-coding RNAs that typically suppress gene expression. Using the next-generation sequencing (NGS) technique (Illumina) and support vector machine classification model, several microRNAs were identified by different groups with the potential to diagnose ADHD (68–72), which required to be tested in future clinical studies to identify the optimal combination. A large epigenome-wide association study (EWAS) discovered several novel epigenetic biomarkers for ADHD from saliva DNA samples, for example, cg17478313 annotated to *SLC7A8* and cg21609804 annotated to Microtubule Affinity Regulating Kinase (MARK)2 that are regulated by single nucleotide polymorphisms (SNPs) (65). Global DNA methylation (GMe) in ADHD patients was lower than in controls (73). One study used a genome-wide DNA methylation assay to analyse blood cells, associating a methylation site on DRD4 with aggressive behavior (9). DNA methylation levels in *LIME1* (cg00446123 and cg20513976) were found to be significantly higher, and those in *SPTBN2* (cg02506324) were significantly lower in children with worse attention performance (74). Using DNA extracted from saliva, Goodman et al. (75) found that altered DNAm at CpGs mapping to *MAD1L1*, *MGC87042*, *PTPRN2*, and *SGK2* were associated with ADHD disorder. Human dopamine transporter (DAT) 5’UTR epigenetic changes and serum antibodies can be further used to confirm ADHD diagnosis and/or to predict the efficacy of treatment (76). Another study analyzing SNP genotype in participants with Spanish origins discovered the genotype of *DCDC2*-*DYX1C1* SNPs presented a significant association with ADHD (77). Spontaneously hypertensive rats are often used to model ADHD, which show reduced startle and repulse inhibition attributed to two quantitative trait loci (QTL), Ofil1 (on chromosome 4) and Ofil2 (on chromosome 7) (59). However, this has not been confirmed in humans. Although positron emission tomography (PET) is an imaging procedure, it uses radioligand to measure the metabolic activity of the tissues. A study using PET to correlate norepinephrine transporter distribution and its DNA methylation (78). They found significant differences in norepinephrine transporter methylation levels at several cytosine-phosphate-guanine (CpG) sites between ADHD and control groups and a negative correlation between methylation of a CpG site at promoter “region 1” and norepinephrine transporter distribution in the thalamus, locus coeruleus, and the raphe nuclei (78). Although brain mapping may be more accurate than the information obtained from the saliva and blood, it needs to be noted that PET is very pricy, which is unlikely to be used in each patient beyond research purposes.

### 2.3. Physiological biomarkers

Physiological biomarkers can also be potentially added to the suite of diagnostic criteria in addition to DSM-5. There were 20 papers that used physiological markers alone or in combination with imagining analysis (Table 2). Eye physiology has been used in several studies (80, 81). One used pupillometry to assess pupil size (80). Pupillometry measures pupil dilation when exposed to a stimulus, specifically focusing on minute fluctuations (80). These studies link ADHD with the dynamics of pupil sizes. Interestingly, at rest, ADHD individuals show large pupil diameters and low temporal complexity and symmetry (81), which was smaller than the controls during spatial working memory tasks (82). One study used a visual cue experiment to show weak eye vergence response linked in adults with ADHD with 79% accuracy (83). Another study associated microsaccades (involuntary, small, jerk-like eye movements with high velocity) with ADHD (98). Eye responses may be used as an objective marker to diagnose ADHD.

Biomarkers in the brain and body fluids may also be used to screen for ADHD in high-risk populations. Using MRI imaging, several neurotransmitters, glutamate, N-Acetyl Aspartate and choline, were found to be reduced in the right prefrontal cortex of ADHD brains (36). A study on saliva associated ADHD with lower levels of bedtime and awakening salivary cortisol levels, high levels of inflammatory markers C-reactive protein and IL-6 while TNF-α and brain-derived neurotrophic factor (BDNF) levels were decreased (84). However, only BDNF was proposed as a potential biomarker for ADHD, as the other proinflammatory markers can be increased in other inflammatory conditions, such as the mouth or upper respiratory tract infections. One study showed that perinatal exposure to Pitocin (injectable oxytocin) use doubles the risk factor for ADHD (85); however, it is not a suitable biomarker as it is mainly used in pregnant mothers during labour. Lead exposure has been suggested as a risk factor for ADHD by epidemiological studies since it is a strong neurotoxin. A study examined the correlation between lead levels in the bone and blood and ADHD scores (86). Higher blood-lead levels correlate with higher hyperactivity/impulsivity scores, while higher bone-lead levels correlate with both higher hyperactivity/impulsivity and oppositional-defiant-disorder scores (86). As this study did not include any control participants, it is unclear whether lead level alone can be used as part of the diagnostic criteria. Metabolites of the kynurenine pathway can interact with brain glutamate receptors, which are proposed to be involved in the development of ADHD (88). A study using liquid chromatography–tandem mass spectrometry (LC–MS/MS) showed markedly reduced blood anthranilic acid (−60%) levels and increased tryptophan (+11.0%) and kynurenine (+48.6%) levels in children with ADHD (88). Compared with controls, lower blood levels of arachidonic acid in ADHD were also reported by other studies (49, 88). Anthranilic acid, kynurenine, Triiodothyronine, dehydroepiandrosterone sulphate, 4-hydroxy-2-nonenal-mercapturic acid, adiponectin, and all major serum sphingomyelins have been reported to be promising blood biomarkers to diagnose ADHD (49, 87–89). Another study on boys with ADHD found a 50% reduction in the binding capacity of muscarinic acetylcholine receptors in fibroblasts due to reduced cholinergic receptor density (93). Upon verification by a larger-scale study, this can be another potential diagnostic biomarker.

Certain chemicals in the patient’s blood or in only the mother’s blood have been suggested to predict the risk of ADHD in their children. A prospective birth cohort study observed a positive association between maternal blood levels of an acetaminophen metabolite measured within 1–3 days postpartum and ADHD diagnosis in offspring (94). Selenium is an essential trace element involved in neurodevelopment. A prospective birth cohort study found a positive association between maternal red blood cell selenium level and the child’s risk of ADHD (95). Another studying using gas chromatography–mass spectrometry (GC–MS) in conjunction with behavioral studies found links between the onset of ADHD in children and maternal blood levels of ethyl glucuronide (a direct metabolite of ethanol) in the third trimester (96). In another study, the hyperactive–impulsive dimension of ADHD was related to both children’s and maternal telomere length, but that of the father (79).

Recently, it has been increasingly recognized that gut microbiota can influence brain function and behaviors *via* the gut-brain axis. A study finds *Bacteroides ovatus* and *Sutterella stercoricanisusing* populations positively correlated to ADHD symptoms (97). Although such biomarkers can be useful in studying the mechanism of ADHD and searching for new treatment strategies, their application in the diagnosis may be limited.

### 2.4. Histological biomarkers

No histological biomarkers in the brain were reported in the papers selected for this review, which is not surprising. This type of marker is normally available from post-mortem studies, which may not be feasible, especially when needed to be combined with a standardized clinical diagnosis compared to the other three categories of biomarkers discussed above.

## 3. Discussion

In this review, several biomarkers from the included studies are promising to be objective diagnostic tools for ADHD once their effectiveness and accuracy can be confirmed with larger cohort studies for ADHD diagnosis. These include imaging methods, biological markers in the body fluids and genome, as well as physiological responses to specialized tests.

EEG and MRI are noninvasive assessments that have been used in a substantial amount of papers to uncover possible biomarkers of ADHD. Brain activities measured by fMRI provide parameters suitable for ADHD diagnosis. For example, during the Switch Task (a test that assesses abilities to turn attention to differing tasks), there was lower activation in bilateral inferior PFC and the left inferior parietal lobe, as well as lower connectivity between right inferior PFC and surrounding brain regions such as the parietal lobes and the basal ganglia (41). White matter radial diffusivity of fewer white matter tracts can be a potential predictor of symptom severity in people with ADHD, specifically between the right inferior frontal gyrus and right superior temporal gyrus in those with cognitive deficits (44). Gray matter volumes are also lower in participants with ADHD in the bilateral hippocampus and right orbitofrontal cortex in MRI scanning, which may contribute to ADHD-like behaviors and cognitive abnormalities (35).

The inferior frontal gyrus has been suggested to be linked to motor response inhibition in the go/no-go tasks (52). Using fNIRS reflecting oxyhemoglobin signals, reduced signaling in the right inferior frontal gyrus was associated with ADHD, which can be used as a tool to determine the treatment efficacy of methylphenidate targeting this area (52). Similar results were found when lowered inferior frontal gyrus/middle frontal gyrus cortical activation was increased by atomoxetine (a non-stimulant drug to treat ADHD) using fNIRS (50). In addition, lower activation of the right and middle PFC and compensatory brain function in the left PFC were found in ADHD participants (46). Interestingly, the hemodynamic shifts in the frontoparietal region assessed by NIRS also led to another potential blood biomarker linked to ADHD, polyunsaturated fatty acids, which however, needs to be verified in large cohort studies due to the small sample size (24 ADHD vs. 22 Control) in the study included here (49).

In studies using EEG, reduced resting state alpha power correlates with cortical hyperactivation in ADHD participants during the continuous performance task (14). P3b, an event-related potential, was also found to be enhanced in ADHD patients when compared to control participants, which was linked to compensatory brain function in the frontoparietal cortex (19). It further suggests using ERP markers of reduced visual short-term memory storage capacity as an ADHD endophenotype (19). In contrast, P3 ERP was shown to be reduced in patients with ADHD (10, 19). It needs to be noted that the sample size in the study by Alexander et al. (10) (*n* = 175 both ADHD and age-matched controls) was more than 10 times that by Weigand et al. (19) (*n* = 16 in each group), suggesting the need for even larger cohort and multiple centre studies to verify the findings. In DSM-5, the last criteria, or criterion ‘E’, states that for one to be diagnosed with ADHD, the symptoms cannot be better explained by another psychiatric disorder. Therefore, when an individual with ADHD also shows any comorbidity (e.g., dyslexia), the accuracy of diagnosis may be reduced. A reduced theta/beta ratio in EEG has also been shown to enhance the accuracy of diagnosing ADHD with the presence of comorbidities from 61% to 88% (26).

Molecular biomarkers often require the collection of blood and/or buccal swabs; however, they may be more accurate in determining the genetic or epigenetic susceptibility and diagnosis of ADHD. Several molecular biomarkers in the papers included in this review are related to the dopaminergic system. In ADHD, the inhibitory mechanism is disrupted due to a lack of dopamine released from the presynaptic neurons. Five studies used DAT1 as a biomarker (58, 60–62, 106). Although the polymorphism of DAT1 is not directly associated with ADHD, however, the 10/10 VNTR of DAT1 genotype correlated with lower processing abilities with poorer performance to focus and short-term memory and attention span (106). Braet et al. (58) also found heterogeneity in response inhibition in individuals with ADHD and the DAT1 phenotype using fMRI. While gene polymorphism plays a significant role in the development of ADHD, epigenetic mechanisms such as DNA methylation which can turn genes on or off, can also be involved in the development of ADHD. For example, methylation of DAT1 promoters has also been found to be associated with the development of ADHD (76). In addition, low anthranilic acid was associated with the risk of ADHD, but this correlation has not been agreed upon by other papers (88). The link between maternal blood level of ethanol metabolite ethyl glucuronide and ADHD behaviors in offspring is only useful as a biomarker if the mother had excessive alcohol consumption during pregnancy, thus it is not useful as a diagnostic tool (96). Epigenetic biomarkers are another type of novel biomarkers, which may also serve as the underlying mechanism for sporadic cases without obvious causes (59, 65, 67, 77). The common issue with human studies searching molecular markers is the sample size, which may be due to the high cost of screening a large number of genes. Although promising as a potential diagnostic tool, large cohort studies are still needed to verify the optimal combination of the biomarkers before a diagnostic kit can be developed. The cost may also be unbearable for most families without government support.

Using physiological markers, such as pupillometric, is noninvasive, easy to set up, and cost-effective, which can be considered by clinicians in the near future in diagnosing ADHD (80). Salivary cortisol levels in combination with BDNF are also noninvasive and easy to collect (84). Their reliability in diagnosing ADHD is again pending further verification by larger cohort studies. Such studies should perhaps be performed by a consortium of experts in basic research and clinical practice to ensure the study design and results are translatable.

Interestingly, most studies only examined one category of diagnostic methods in the literature. Only a couple of research teams have included two categories of biomarkers in their studies, and attempted to correlate the limited variables in these two categories. This may be due to the limitations of the time and cost of performing such studies on the same cohort of participants, which makes it even more difficult for a longitudinal type of study design. Nevertheless, when resources are available, cross-disciplinary teams can consider adopting several categories of biomarkers, which may increase the diagnostic accuracy of the biomarkers.

There are also some limitations in this review itself. Firstly, we only searched three commonly used databases, PubMed, Ovid Medline, and Web of Science, as these cover the majority of journals. However, new journals can not be listed in these databases, and it is possible important studies were missed. Future updates on this topic can include additional search engines, such as Google Scholar. The search was performed before August 2022, therefore new papers published afterwards were not included. Secondly, we only included papers which identified biomarkers and therefore were positive in the study outcomes. Here, we summarized the positive results which are synonymous with the notion of a biomarker (i.e., if you measure something that is not associated with a disease, it is not a biomarker of that disease). We did not want to overinterpret studies which have measured clinical or biological factors but have not assessed these as being biomarkers. Therefore, we selected the information that would be potentially useful clinically. We did not list the papers according to ADHD subtypes or age groups. However, we did include the age of the participants in Table 2. Most study designs have small cohort sizes, therefore, limiting our ability to subtype the patients. However, in the field, the observation that a factor is evaluated or a clinical trait is identified is still useful information, even if it is not a specific biomarker for ADHD. This review can serve as a reference to suggest the design of future clinical trials which need to be carried out in multiple centers to confirm the validity of the potential markers.

## 4. Conclusion

There are several promising biomarkers that can be further researched in larger cohort studies for ADHD diagnosis. Future studies can include participants with diverse ethnic and cultural backgrounds in different countries, as well as compare sex differences in the reliability of these biomarkers for ADHD.

## Author contributions

HC and BO designed the study and prepared the first draft. YY, DO, SW, and CY performed the literature search. All authros contributed to the writing of the manuscript, and reviewed the final manuscript. All authors have agreed to the published version of the manuscript.

## Funding

This work was supported by grants from Australian National Health and Medical Research Council (APP1158186), the National Natural Science Foundation of China (NSFC 81971309, 32170980 and 82201577), Guangdong Basic and Applied Basic Research Foundation (2022B1515020012), China Postdoctoral Science Foundation (2022M723649), and Shenzhen Fundamental Research Program (JCYJ20190809161405495, JCYJ20210324123212035, JCYJ202205303001572, and RCYX20200714114644167).

## Conflict of interest

The authors declare that the research was conducted in the absence of any commercial or financial relationships that could be construed as a potential conflict of interest.

## Publisher’s note

All claims expressed in this article are solely those of the authors and do not necessarily represent those of their affiliated organizations, or those of the publisher, the editors and the reviewers. Any product that may be evaluated in this article, or claim that may be made by its manufacturer, is not guaranteed or endorsed by the publisher.

## Abbreviations

ADHD: attention deficit hyperactivity disorder
BDNF: brain derived neurotrophic factor
“COS”: complementary on the opposite strand
DAT1: dopamine transporter
EEG: electroencephalography
ELISA: enzyme linked immunosorbent assay
ERP: event related potential
fMRI: functional magnetic resonance imaging
fNIRS: functional near-infrared spectroscopy
HNE-MA: 4-hydroxy-2-nonenal-mercapturic acid
IFG/MFG: inferior and middle prefrontal gyri
LC–MS: Liquid Chromatography-Mass Spectrometry
LC–MS/MS: liquid chromatography–tandem mass spectrometry
MS: Mass spectrometry
MRS: magnetic resonance spectroscopy
MRI: magnetic resonance imaging
N/A: Not Applicable
NGS: next-generation sequencing
NIRS: near-infrared spectroscopy
oxy-Hb: oxygenated hemoglobin
PFC: prefrontal cortex
pTMS: paired-pulse transcranial magnetic stimulation
rsEEG: resting-state EEG
RST: reverse Stroop task
SICI: short interval cortical inhibition
SVM: support vector machine
RT-PCR: reverse transcription-polymerase chain reaction
TMS: transcranial magnetic stimulation
UTR: untranslated region
PET: positron emission computed tomography
